# 

**Published:** 1894-09

**Authors:** 


					CONTENTS.
The Bristol Meeting of the British
Medical Association, 1834
161
Ergot of Rye as an Oxytocic.
By J.
(j. Swayne, M.D. Lond
223
Case of Myxcedema.
By W. A. Hayes,
M.R.C.S. Eng., L.R.C.P. Loud.
230
Two Cysts of Unusual Origin.
By
Charles A. Morton, F.R.C.S. Eng
232
Progress of the Medical Sciences:
Medicine.
By George Parker,
M.D.
Surgery.
By W. H. Harsant
Dermatology.
By Henry Waldo,
M.D.
Reviews of Books
230
Notes on Preparations for the Sick
283
The Library of the Bristol Medico-
Chirurgical Society  268
Scraps
271

				

